# Young Adult Carers during the Pandemic: The Effects of Parental Illness and Other Ill Family Members on COVID-19-Related and General Mental Health Outcomes

**DOI:** 10.3390/ijerph19063391

**Published:** 2022-03-13

**Authors:** Giulia Landi, Kenneth I. Pakenham, Silvana Grandi, Eliana Tossani

**Affiliations:** 1 Department of Psychology “Renzo Canestrari”, University of Bologna, 40127 Bologna, Italy; silvana.grandi@unibo.it (S.G.); eliana.tossani2@unibo.it (E.T.); 2 Laboratory of Psychosomatics and Clinimetrics, Department of Psychology, University of Bologna, 47521 Cesena, Italy; 3 School of Psychology, The University of Queensland, Brisbane, QLD 4072, Australia; k.pakenham@psy.uq.edu.au

**Keywords:** young adult carers, emerging adulthood, parental illness, pandemic, mental health, wellbeing

## Abstract

The mental health impacts of the COVID-19 pandemic on young adult carers have been neglected. This study aimed to identify COVID-19 related risk factors for young adult carers and to investigate their mental health relative to non-carer peers. Of the 1823 Italians aged 18–29 who completed an online survey, 1458 reported no ill family member (non-carers). Young adult carers included 268 with an ill parent, and 97 with an ill non-parent family member. Two mental health outcome categories were measured: COVID-19-related (risky health behaviors, loneliness, home violence, fear of COVID-19) and general (anxiety, depression, wellbeing). Six COVID-19 related risk factors were significantly correlated with poorer mental health in young adult carers. These factors constituted a COVID-19 Context Index. Compared to non-carers, young adult carers reported poorer mental health across all outcomes, as expected. The prediction that young adult carers caring for an ill parent would report poorer mental health than those caring for ill non-parent family members was evident only for the COVID-19-related mental health outcomes. The elevated rates of clinically significant distress and pandemic-related mental health problems among young adult carers highlight this group as a priority for mental health promotion interventions and whole-of-family support across multiple sectors.

## 1. Introduction

A substantial body of published international research provides evidence on the adverse psychosocial impacts on youth caring for a family member, particularly a parent, who has a serious mental or physical health condition [1,2,3,4]. Most studies in this field have investigated children and adolescents up to the age of 18 years (referred to as young carers), with markedly less research on young adult carers (18–24 years) [5]. Yet the young adult developmental phase (also referred to as emerging adulthood) involves critically important milestones such as increasing autonomy, identity formation, career development, and navigating intimate relationships [6]. Negotiating these milestones may be disrupted by the demands of caring for a family member. Indeed, a recent review of young adult carer research found that their caregiving is associated with adverse impacts on physical and mental health, personal development, and decision-making regarding future career, education, and financial and social prospects [5]. Evidence suggests that the caregiving demands of informal carers have been exacerbated by the COVID-19 pandemic disruptions to health care delivery and the associated unprecedented levels of uncertainty both for the people they care for and for their own health and wellbeing. However, to our knowledge there is no published quantitative research on the effects of the pandemic on young adult carers specifically. Hence, the purpose of the present study is to identify COVID-19 and lockdown mental health risk factors for young adult carers and to investigate the mental health of young adult carers relative to young adults who are not carers in the context of the COVID-19 pandemic.

For many young adults, the family caregiving situation can dominate their daily living and lead to significant restrictions and disruptions including: less time for hobby and leisure pursuits and social activities with peers, interruptions to schooling or work, worry about the ill family member, strained family relations, tension with respect to balancing the demands of establishing a career with those of caregiving, and decision-making conflicts regarding future life planning relative to their ongoing caregiving predicament [7,8,9]. These difficulties are exacerbated by the tendency for young adult carers not to disclose their caregiving situation to others, in part, because of the stigma associated with informal caregiving [10]. These challenges, in addition to those arising from the caregiving itself, can interfere with the timely and smooth navigation of emerging adulthood milestones and lead to mental health problems.

Regarding the mental health impacts of young adult caregiving, a recent scoping review of 12 studies found evidence of adverse effects on emotional functioning and future development [5]. In addition, a review of research on young adult higher education student carers of an elderly family member identified six studies that fitted this narrower focus [10]. Across both reviews, only two quantitative studies had examined mental health using standardized measures and compared young adult carers to non-carers [5]. Greene et al. [11] surveyed US university students and found that young adult carers of a family member reported significantly higher levels of depression and anxiety symptoms than non-carers. Similarly, in a study of Norwegian university students, those who cared for an ill family member reported more negative health outcomes (anxiety, depressive and somatic symptoms, and sleep problems) compared to non-caregiving students [8]. This latter study also found that higher levels of caregiving were related to increases in mental health problems [8]. Despite these adverse impacts, young adult carers have also reported some positive effects of caregiving, such as the acquisition of organizational skills [9].

Given the widely documented adverse social and mental health impacts of the COVID-19 pandemic and lockdowns on individuals [12,13,14,15] and families [16], the negative effects of informal caregiving are likely to be intensified and the mental health inequalities between young adult carers and non-carer peers amplified [17]. Indeed, one study found that a sample of UK informal adult carers, which included young adults, reported higher levels of depressive symptoms and anxiety than non-carers across five time points over seven months of the pandemic in 2020 [18]. A study of Austrian adult informal carers, which also included young adults, found that the pre-pandemic gap between lower and higher psychological wellbeing in carers and non-carers respectively, had increased three months into the pandemic [19]. Given the lack of quantitative data on the mental health of young adult carers relative to non-carer peers and the absence of such data during the COVID-19 pandemic, the present study addresses this research gap.

We located one published study providing data on COVID-19 and lockdown factors that appear to adversely affect the mental health of young carers [20]. This study collected qualitative data via interviews with 20 UK young carers, including five young adults, recruited from young carer organizations. The semi-structured interview was designed to investigate participants’ experiences of the pandemic. Descriptive quantitative data was also collected via an online survey from a larger sample of young carers (*n* = 177); however, no information was provided on the characteristics of the survey sample or of the measures used. Both qualitative and quantitative findings showed that lockdown restrictions and the anxiety related to COVID-19 infection were central to their difficulties [20]. Qualitative data showed that the pandemic had intensified their caregiving responsibilities and demands, disrupted education and learning, increased their time spent with the care-recipient, evoked concerns about their care-recipient’s vulnerability to COVID-19 infection, and increased their difficulties in accessing formal and informal support for themselves and the care-recipient due to social distancing restrictions [20]. Survey data showed that 78% of respondents reported feeling lonely or isolated, and 74% reported that their mental health had been adversely impacted by the pandemic. It is therefore important to identify the specific COVID-19 and lockdown factors that place young adult carers at increased risk of experiencing the adverse mental health impacts of the pandemic [17].

A potentially important factor that is likely to influence the level of caregiving strain a young adult carer experiences is the nature of the family connection to the care-recipient. The literature on young adults indicates that to a certain degree most youths perform caregiving tasks and assume some responsibility for contributing to family functioning. Youth caregiving has been viewed as occurring on a continuum with basic household chores often undertaken at the lower end and at the higher end the assumption of caregiving responsibilities and activities at the expense of important developmental milestones [21,22,23,24]. Hence, youth caregiving is applicable to diverse contexts, although its extent is likely to be intensified by illness in a family member, especially in parents [23,24,25,26]. For example, given that parents typically assume the caregiving role in relation to their children, a reversal of this role is likely to create more challenges than caring for another family member. This reversal of roles within the family system, whereby the child is acting as a parent or as a ‘mate’ to its parent, is called parentification. One study showed that young adult carers reported higher levels of parentification [27] than non-carer peers, though this study did not compare young adult carers of a parent with young adult carers of other family members. Findings from a largescale survey of Australian young carers, which included young adult carers, showed that those who cared for an ill parent reported more mental health problems [26] and caregiving responsibilities [24] than young carers of other ill family members. However, no published research has examined whether similar effects are apparent in samples of young adult carers that exclude young carers. Hence, in the present study we examine whether there are variations in young adult carers’ mental health associated with caring for an ill parent vs. another ill family member.

### The Present Study

The purpose of the present study is to add to the limited knowledge base on the mental health impacts of family caregiving on young adult carers and to investigate the mental health status of this neglected group of informal carers in the context of the COVID-19 pandemic. To this effect, the study has two aims. The first aim is to identify COVID-19 and lockdown contextual elements that constitute mental health risk factors for young adult carers in both parental illness and other ill family member caregiving contexts. The second aim is to explore differences in COVID-19-related mental health and general mental health outcomes across three groups of young adults: carers in a parental illness context, those caring for an ill non-parent family member, and non-carers. Informed by the prior research reviewed above, we hypothesized that, after controlling for relevant socio-demographics and COVID-19 related risk factors, young adult carers caring for an ill parent will report poorer mental health than young adult carers of other ill family members and that these two groups, in turn, will report poorer mental health than non-carer peers.

## 2. Materials and Methods

### 2.1. Participants and Recruitment Procedure

A total of 1823 young adults aged 18–29 completed an online survey between 19 January 2021 and 2 February 2021 during the second Italian mandatory lockdown. Most of the studies on young adult carers have recruited from higher education student populations or from organizations that support formally identified young carers and young adult carers. In the present study, we recruited young adults from the general community, with no requirement for participants to self-identify as a carer. Regarding the latter, given the evidence that young people in caregiving roles do not always identify as carers [23,28], investigating only those who self-identify as carers may exclude young people who have substantial care responsibilities but do not identify as a carer. Although most research on young adult carers has focused on the 18–24 years age range, we used the broader 18–29 range in this study. There is widely published data showing that in high-income countries emerging adulthood typically extends up to age 29, with parallel trends towards longer time in education and later entry into marriage and parenthood [6]. This revised extended developmental period for emerging adulthood is now widely used (e.g., see review [29]).

**Figure 1:**
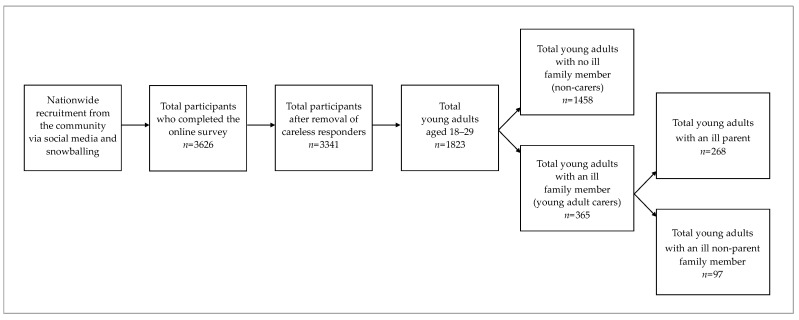
Flow chart depicting participant selection into this study.

At the time of data collection, most Italian Regions had been classified in the previous two months into one of two risk categories: red zone (i.e., same restrictions as in the first 2020 strict national lockdown) or orange zone (i.e., people could go to work, but most non-essential industries were closed, and people could not leave their province). Data was drawn from a larger COVID-19 mental health project: inclusion criteria were ≥18 years and being resident in Italy. Recruitment was conducted through social media and a snowballing approach, whereby participants invited friends and acquaintances to participate in the study. The survey was advertised as research designed to investigate protective psychological resources during the COVID-19 pandemic. The survey was developed on Qualtrics software and took approximately 20 min to complete. For the present study, we selected participants aged 18–29 years from the total sample for the larger project. It was not possible to calculate an accurate response rate because recruitment was primarily conducted through social networks. A total of 3626 participants completed the online questionnaire. In completing online surveys some participants can ‘randomly’ select a response to items or respond without reflecting on the content of the items [30] and are referred to as ‘careless responders’. To identify careless responders, we included two attention check items in the middle and towards the end of the survey: “To demonstrate your attention, select the answer agree a little”, and “To demonstrate your attention, select the answer never.” Careless responders were identified as those who answered incorrectly to one or both attention check items and they were excluded from the sample. A flow chart depicting participant selection into this study is presented in Figure 1. This study was approved by the Ethics Committee of the University of Bologna.

### 2.2. Measures

#### 2.2.1. Socio-Demographics

Participants reported their gender, age, education, marital status, occupation, socio-economic status, nationality, and presence of a physical health condition.

#### 2.2.2. Family Caregiving Status

Participants reported whether any person they were living with had a serious physical or mental health condition. If ‘yes’, they indicated who was ill (either a parent or another ill family member). Two variables were subsequently created: parental illness (0 = no and 1 = yes) and other ill family member (0 = no and 1 = yes). As summarised in Figure 1, 79.98% of participants (*n* = 1458) reported no family member with a serious health condition (labelled the non-carer group), and 20.02% (*n* = 365) reported an ill family member in their household. Of the latter, 14.70% (*n* = 268) reported living with a parent with a serious health condition (labelled the PI group), and 5.32% (*n* = 97) reported living with a non-parent family member with a serious health condition (labelled the OIFM group). Other ill family members comprised grandparents (*n* = 102, 5.60%), siblings (*n* = 66, 3.62%), with the remainder reporting some other ill family member (i.e., uncles/aunts or cousins).

#### 2.2.3. COVID-19 and Lockdown Context Variables

The following 11 variables were assessed to obtain information on participants’ COVID-19 and lockdown experiences: (1) participants’ perceptions of the adequacy of home space during the mandatory restrictions was evaluated with the following item rated on a 5-point scale (0 = not at all to 4 = very much): “Is the size of your home insufficient to guarantee your personal space, despite the mandatory lockdown, such as number of rooms in relation to the people you live with?” (2) Participants also rated the amount of time they spent working from home (‘smart working’) or in distance learning from the beginning of the second lockdown on a 5-point scale (0 = never in smart working or distance learning to 4 = always in smart working or distance learning). In addition, participants reported whether they were (3) currently living in a red-zone, whether they were (4) infected by COVID-19, or (5) hospitalized, as well as (6) whether family members were infected, (7) hospitalized, or (8) deceased due to COVID-19. Finally, participants indicated whether they had (9) lost work, (10) were receiving a redundancy payment or (11) if their income had substantially decreased due to COVID-19 and lockdown restrictions.

#### 2.2.4. COVID-19-Related Mental Health Outcomes

##### Risky Health Behaviors

Participants rated on a 5-point scale (0 = no difficulty to 4 = extreme difficulty) the extent to which they noticed during the second lockdown increases in the frequency of risky health behaviors typically observed during lockdowns [31,32]: (1) alcohol use, (2) drug use, (3) use of tobacco or electronic cigarettes, (4) gambling, (5) taking more medications than prescribed, (6) consumption of sweets and/or salty snacks between main meals, (7) watched the news, listened to the radio or surfed the internet for information on COVID-19. In order to create an index of risky health behaviors, we first inspected the skewness and kurtosis of these seven items. The drug use and gambling items evidenced high skewness (4.04 and 5.29, respectively) and kurtosis values (18.28 and 30.78, respectively), and were removed. Subsequently, we inspected correlations between the remaining five items and the COVID-19 and lockdown context variables performed on the total young adult carer subsample. Only one of the remaining five risky health behaviour items was unrelated to all COVID-19 and lockdown context variables (use of tobacco or electronic cigarettes). The remaining four risky health behaviour items were significantly correlated with one or more of the COVID-19 and lockdown context variables: alcohol use (insufficient home dimension *r* = 0.124, *p* < 0.05), medication overuse (family member COVID-19 death, *r* = 0.20, *p* < 0.01), snacking (working or studying from home, *r* = 0.123, *p* < 0.05), and problematic levels of seeking COVID-19 information (family member COVID-19 death, *r* = 0.104, *p* < 0.05). These four risky health behaviour items constituted the risky health behaviors index. The mean of ratings across these four items was calculated. Higher scores reflect an increase in risky health behaviors during the second lockdown (range 0–4).

##### Loneliness

Loneliness was assessed with the validated Italian version [33] of the short form of the UCLA Loneliness Scale developed specifically for use in large-scale surveys [34]. Participants reported how often they “felt that they lacked companionship,” “felt isolated,” and “felt left out” on a 4-point Likert scale (0 = hardly ever or never to 3 = very often). Scores were averaged and higher scores indicated greater loneliness (range 0–3). The instrument has been shown to have good psychometric properties [34]. The observed Cronbach’s alpha was 0.87.

##### Increase in Home Violence

To gauge participant’s perceptions of increases in domestic violence, the following item was rated on a 5-point scale (0 = not at all to 4 = very much): “Have verbal and/or physical violent behaviors increased at home during the current mandatory lockdown?”. Higher scores indicate increases in domestic violence during lockdown.

##### Fear of COVID-19

Fear of COVID-19 was measured by the validated Italian version [35] of the 7-item Fear of COVID-19 Scale [36]. This scale measures emotional, cognitive, physiological, and behavioral manifestations of fear related to COVID-19. Each item (e.g., “It makes me uncomfortable to think about coronavirus-19”) is rated on a 5-point Likert scale (1 = strongly disagree to 5 = strongly agree). Item scores are summed, with higher scores reflecting higher fear of COVID-19 (range 7–35). Previous studies have reported good psychometric properties [36]. The observed Cronbach’s alpha was 0.83.

#### 2.2.5. General Mental Health Outcomes

##### Anxiety

Anxiety was assessed with the validated Italian version [37] of the General Anxiety Disorder Scale [38]. This questionnaire measures anxiety symptoms over the past two weeks. Items are rated on a 4-point Likert scale (0 = not at all to 3 = nearly every day). Item scores are summed, with higher scores reflecting higher anxiety. The instrument has been shown to be psychometrically sound [39]. The observed Cronbach’s alpha was 0.90. Normative data provide the following ranges for the total score: minimal (0–4), mild (5–9), moderate (10–14), and severe (15–21) anxiety symptoms [38].

##### Depression

The validated Italian version [40] of the Patient Health Questionnaire-9 (PHQ-9) [41] was used to measure depressive symptomatology over the past two weeks. Items are rated on a 4-point Likert scale (0 = not at all to 3 = nearly every day). All item scores are summed, with higher scores indicating higher depression. Normative data provide the following ranges for the total score: normal (0–4), mild (5–9), moderate (10–14), moderately severe (15–19), and severe (20–27) depressive symptoms. The measure has demonstrated sound psychometric properties [42]. The observed Cronbach’s alpha was 0.88.

##### Wellbeing

The validated Italian version [43] of the Mental Health Continuum Short Form [44] was used to assess wellbeing. This 14-item scale evaluates emotional (i.e., “How often did you feel happy?”; 3 items), psychological (i.e., “How often did you feel good at managing the responsibilities of your daily life?”; 6 items), and social (i.e., “How often did you feel that you belonged to a community?”; 5 items) wellbeing. Items are rated on a 6-point Likert scale (0 = never to 6 = every day) with the last month as the timeframe. Items were summed with higher scores indicating higher wellbeing. According to normative data, wellbeing status is classified in the following categories: flourishing or high levels of wellbeing (ratings of 4 = almost every day or 5 = every day on at least one item of the emotional wellbeing scale, plus ratings of 4 or 5 on at least six of the eleven items of the psychological and social wellbeing scales combined), languishing or the absence of wellbeing (ratings of 0 = never or 1 = once or twice on at least one item of the emotional wellbeing scale, plus ratings of 0 or 1 on at least six of the eleven items of the psychological and social wellbeing scales combined), and moderate wellbeing (ratings falling between the flourishing and languishing categories of wellbeing) [45]. The measure has demonstrated sound psychometric properties [46]. The observed Cronbach’s alpha was 0.91.

### 2.3. Data Analysis Approach

Descriptive statistics, reliabilities, and correlations among study variables were carried out in IBM SPSS 24. All other analyses were conducted in Mplus 8.4 with the robust maximum likelihood estimator (MLR) [47]. The overall percentage of missing data was 3.16%. Little’s [48] Missing Completely at Random test on the variables of interest yielded a normed χ^2^ (χ^2^/df) of 0.01. This index, which can be used to correct for sensitivity of the χ^2^ for large samples [49], is low and suggests that data are missing completely at random. Therefore, the Full Information Maximum Likelihood estimator was used to handle missing data.

Regarding preliminary analyses, we examined descriptive data on socio-demographics and the COVID-19 and lockdown context variables. To investigate whether the three groups differed on socio-demographics, we conducted ANOVAs on continuous variables and chi-square tests on categorical socio-demographics. We compared data on the anxiety, depression, and wellbeing measures with normative data in each of the groups (PI, OIFM, non-carer) to identify variations in the proportion of young adult carers relative to non-carers who reported clinically significant levels of distress and markedly low levels of wellbeing.

To address the first aim, we conducted correlations between COVID-19 and lockdown contextual factors and the dependent variables (i.e., COVID-19-related mental health and general mental health outcomes) in each of the three groups (PI, OIFM, and non-carer) to identify COVID-19 and lockdown risk factors in young adult carers. We inspected whether significant correlation coefficients in the non-carer group differed from the corresponding significant or non-significant correlation coefficients in the PI or OIFM groups. These comparisons between pairs of correlation coefficients were performed using the Fisher’s z-test with Bonferroni correction [50]. In this procedure, correlation coefficients are transformed using Fisher’s Z transformations, so that Z has an approximate normal distribution. The null hypothesis that two correlations coefficients from separate samples are equal is tested by adjusting for the unequal sample sizes of these groups.

To address the second aim, we conducted two multivariate linear regressions in order to examine differences in COVID-19-related mental health and general mental health outcomes across the three groups. The endogenous variables were the COVID-19-related mental health outcomes (i.e., risky health behaviors, loneliness, increase in home violence, and fear of COVID-19) and the general mental health outcomes (i.e., anxiety, depression, and wellbeing) in the first and second set of regressions, respectively, while the exogenous variables were the two family caregiving status dummy variables (i.e., PI and OFMI). Because the non-carer group was represented as a score of 0 on both dummy variables, the regression intercept equaled the mean score on the outcome variable for the non-carer group. The regression coefficients for the PI and OFMI dummies provide a test of the mean differences between the two young adult carer groups and non-carers, while controlling for the presence of OFMI or PI, respectively. Multivariate analyses controlled for sociodemographic variables that significantly differed among the three groups as well as for COVID-19 context and lockdown variables. Local effect size in multiple regressions was calculated with Cohen’s ƒ^2^ [51], with effect sizes of 0.02, 0.15, and 0.35 considered as small, medium, and large effects, respectively [52].

## 3. Results

### 3.1. Preliminary Analyses

#### 3.1.1. Sample Characteristics

Descriptive data on demographics, caregiving context and COVID-19 context variables in the PI, OIFM, and non-carer groups are depicted in Table 1. The total sample consisted of 1823 young adults (71.75% female; *M*_age_ = 24.41, *SD*_age_ = 2.84). Almost half of the sample (49.15%) reported having a bachelor’s degree, 43.06% had completed secondary school and 3.73% primary school levels, and 4.06% had completed a postgraduate course. Most participants (88.65%) reported being single, while 11.19% were married or living with a partner. More than half of the participants were currently studying (57.98%), 35.38% were currently working, and 9.54% reported not being in education, employment, or training. Regarding socio-economic status, 17.01% reported a mean income lower than €15,000, 42.79% a mean income between €15,001–€36,000, 29.57% a mean income between €36,000–€70,000, and 7.35% above €70,000. Almost all young adults (97.92%) were of Italian nationality. Nine percent of participants reported having a physical health condition.

As for COVID-19 lockdown and context variables, participants reported a mean rating of 1.41 (*SD* = 1.03, range 0–4) for insufficient home space during lockdown, and a mean rating of 2.34 (*SD* = 1.85, range 0–4) in the amount of time spent working from home or in distance learning. Most participants (70.43%) reported currently living in an orange zone, while 9.27% were in a red zone. A total of 14% of participants declared having been infected with COVID-19, with none requiring hospitalization. Regarding family members, 24.51% had been infected with COVID-19, 4.28% required hospitalization, and 4.00% died because of COVID-19. Finally, 3.24% of participants reported having lost work, 1.97% received a redundancy payment, and 4.17% reported a substantial reduction in income due to the COVID-19 restrictions.

The only significant differences in socio-demographic variables across the three groups (PI, OIFM, non-carers) were as follows: age, *F* (2, 1820) = 3.95, *p* = 0.019, currently working, χ^2^(2, 1823) *=* 17.93, *p* = 0.000, and currently studying, χ^2^(2, 1823) *=* 9.27, *p* = 0.010. In particular, compared to the non-carer group, the PI group was younger (PI *M*_age_= 24.03, non-carers *M*_age_ = 24.50), had fewer participants currently working (PI 25.00%, non-carers 37.72%) and had more participants currently studying (PI 65.67%, non-carers 56.24%). Therefore, we controlled for these variables in multivariate analyses.

#### 3.1.2. Mental Health Descriptive Data

The percentage of participants in each of the three groups (PI, OIFM, non-carer) that fell into the normative bands for the anxiety, depression, and wellbeing measures are reported in Table 2. Relative to the non-carers, a greater proportion of participants in the PI and OIFM groups reported severe levels of anxiety (PI 17.24%, OIFM 18.95% vs. 11.14% non-carers) and depressive symptoms (PI 29.89%, OIFM 27.37% vs. 17.32% non-carers). However, an oppositive pattern emerged for wellbeing. Relative to non-carers, higher proportions of participants in the PI and OIFM groups reported flourishing (PI 27.64%, OIFM 30.85%, vs. non-carers 18.28%) and fewer fell into the languishing band (PI 18.29%, OIFM 19.15% vs. 25.04% non-carers). The differences among the groups are less marked with respect to the mild or moderate ranges for anxiety and depressive symptoms and for moderate levels of wellbeing.

### 3.2. Correlations between COVID-19 Context Variables and Mental Health Outcomes

Correlations between COVID-19 context variables and mental health outcomes in the PI group, OIFM group, and non-carer group are displayed in Table 3.

Of the 11 COVID-19 and lockdown context variables, the item hospitalized due to COVID-19 was removed because no participant endorsed this item. In addition, few participants (1–5%) endorsed the following three items: lost work, receiving a redundancy payment, and substantial income reduction. Due to the lack of response variation and the overlapping theme of reduced income across these three items, they were combined into a single item. Endorsement of one or more of the three items was scored as 1 = yes. No endorsement of all three items was scored as 0 = no. This revised item is referred to as reduced income from hereon and had the following endorsement frequencies: PI 11.57%, OIFM 7.22%, and non-carers 8.44%. Correlations between the remaining eight COVID-19 and lockdown context variables and the COVID-19-related and general mental health outcomes were inspected for each of the three groups in order to identify COVID-19 and lockdown contextual elements that constitute mental health risk factors for young adult carers (see Table 3). Inspection of correlations across the three groups shows that seven out of the eight COVID-19 and lockdown context variables were significantly correlated with one or more of the COVID-19-related and general mental health outcomes, indicating that poorer mental health was related to insufficient home space, working or studying from home, currently living in a red-zone, infected with COVID-19, family member infected with COVID-19, family member deceased due to COVID-19, and reduced income. Family member hospitalized was the only COVID-19 context variable unrelated to all mental health outcomes across the three groups.

To address the first study aim, we identified COVID-19 and lockdown variables that were significant correlates of mental health outcomes among young adult carers only. Of the seven COVID-19 and lockdown context variables significantly correlated with one or more of the mental health outcomes in one or more of the three groups (mentioned above), six emerged as significant correlates of one or more mental health outcomes in at least one of the young adult carer groups: insufficient home space, currently in red zone, infected with COVID-19, family member infected with COVID-19, family member death due to COVID-19, and reduced income.

To ascertain whether a significant correlation coefficient in the non-carer group significantly differed from the corresponding significant or non-significant correlation coefficient in the other two groups (i.e., PI or OIFM), we used the Fisher’s z-test with Bonferroni correction (see Table 3). Results indicated that the only significant differences within pairs of correlation coefficients between the PI and non-carer groups were the following: correlations between currently in red zone and anxiety, depression, and wellbeing. Specifically, currently in a red zone was associated with lower anxiety, depression, and higher wellbeing in the PI group, while the opposite pattern emerged in the non-carer group. One pair of correlation coefficients significantly differed between the OIFM and non-carer groups: higher levels of insufficient home space were correlated with greater fear of COVID-19 in the OIFM group, but the correlation between these two variables was not significant in the non-carer group. The remaining significant correlations between COVID-19 and lockdown context variables and the mental health outcomes in the non-carer group did not significantly differ from the corresponding pairs of non-significant correlation coefficients in either of the young carer groups.

We developed an index of COVID-19 and lockdown risk factors using a procedure identical to that used in the development of a similar COVID-19 risk index [13], and similar to that used in the development of a psychosocial stress index [53]. Both of these indexes consisted of items that had categorical and Likert scale ratings. We selected the six COVID-19 and lockdown context variables that were significantly related to one or more COVID-19-related mental health and general mental health outcomes in the young adult carer groups. These six variables constituted the COVID-19 pandemic and lockdown index of risk factors referred to as the COVID-19 Context Index from hereon. It included one continuous item, insufficient home space (rated on a 5-point Likert scale, 0 = not at all, to 4 = very much) and five dichotomous items which were scored as 0 (no) and 1 (yes, indicative of presence of COVID-19 and lockdown risk factor): (1) currently living in a red-zone, (2) infected with COVID-19, (3) family members infected with COVID-19, (4) family member deceased due to COVID-19, (5) reduced income. The final COVID-19 Index was calculated by summing across the continuous item and five dichotomous items. Higher COVID-19 Context Index scores reflect greater COVID-19 and lockdown risk factors (range 0–7).

Correlations between the COVID-19 Context Index and the mental health outcomes are presented in Table 3. Results showed that the COVID-19 Context Index was not significantly correlated with any outcome for the PI group. However, higher scores on the COVID-19 Context Index were related to poorer mental health across five of the seven outcomes for the non-carer group and across all but one outcome (increase in home violence) for the OIFM group. Significant correlation coefficients ranged from 0.003 to 0.417. Results of the Fisher’s z-test showed that the significant correlation between COVID-19 Context Index and fear of COVID-19 in the OIFM group significantly differed from the corresponding non-significant correlation in the non-carer group. There were no significant differences between all the other corresponding pairs of correlation coefficients between the non-carer group and the PI and OIFM groups that were examined.

### 3.3. Differences in Mental Health Outcomes among Groups

Two sets of multivariate linear regressions were conducted to explore differences in the COVID-19-related and general mental health outcomes across the three groups. Multivariate regressions controlled for the COVID-19 Context Index and the three socio-demographics that significantly differed across groups (i.e., age, currently studying, and currently working). Standardized beta coefficients and Cohen’s ƒ^2^ for each mental health outcome are displayed in Table 4. Results of the first set of multivariate linear regressions showed that, compared to non-carers, the PI group displayed poorer COVID-19-related mental health outcomes (risky health behaviors *β* = 0.052, *p* < 0.05, loneliness *β* = 0.073, *p* < 0.01, increase in home violence *β* = 0.161, *p* < 0.001, fear of COVID-19 *β* = 0.067, *p* < 0.01). In contrast, compared to non-carers, the OIFM group displayed poorer COVID-19-related mental health outcomes in one domain, increase in home violence (*β* = 0.070, *p* < 0.01). The effect of the PI group was significantly stronger than the effect of the OFMI group for increase in home violence with a medium effect for PI (Cohen’s ƒ^2^ = 0.027) compared to a small effect for OIFM (Cohen’s ƒ^2^ = 0.005).

Results of the second set of multivariate linear regressions indicated that, compared to non-carers, both the PI group and the OIFM group reported significantly poorer general mental health across all three outcomes (anxiety PI *β* = 0.114, *p* < 0.001, OIFM *β* = 0.068, *p* < 0.01; depression PI *β* = 0.125, *p* < 0.001, OIFM *β* = 0.078, *p* < 0.01; wellbeing PI *β* = −0.085, *p* < 0.001; OIFM *β* = −0.052, *p* < 0.05). For the three general mental health outcomes, the effect of the PI group was stronger than that of the OFMI group, but effect sizes were uniformly small in both groups (PI Cohen’s ƒ^2^ ranges: 0.007–0.016, OIFM Cohen’s ƒ^2^ ranges: 0.003–0.006).

## 4. Discussion

The purpose of this study was to identify COVID-19 and lockdown mental health risk factors for young adult carers and to investigate the mental health of young adult carers relative to non-carer peers in the context of the COVID-19 pandemic. Six COVID-19 and lockdown risk factors were significantly correlated with poorer mental health on one or more of the COVID-19-related and general mental health outcomes in young adult carers. As predicted, compared to non-carers, the young adult carers reported poorer mental health across all COVID-19-related and general mental health outcomes. The prediction that young adult carers caring for an ill parent would report poorer mental health than young adult carers of ill non-parent family members was most evident for the COVID-19-related mental health outcomes.

Six COVID-19 and lockdown risk factors emerged as significant correlates of poorer mental health in at least one of the young adult carer groups: insufficient home space, currently in red zone, current COVID-19 infection, family member infected with COVID-19, family member death due to COVID-19, and reduced income. More COVID-19 and lockdown risk factors were significantly related to mental health outcomes in the non-carer group because of the markedly larger sample size, and all of the significant correlation coefficients for the non-carer group were of a relatively low magnitude. However, across all three groups significant correlation coefficients were weak ranging from 0.056 to 0.317 with most ≤20.

When we compared all significant correlations between a risk factor and a mental health outcome in the non-carer group with the corresponding correlations in the two young adult carer groups, we found several significant differences. Comparisons involving the PI group showed that being in a red zone was associated with lower anxiety, depression, and higher wellbeing for those in the PI group, while the opposite pattern emerged in the non-carer group. This pattern of findings may be due to the young adult carers of an ill parent having the perception that red zone restrictions would protect them and their care-recipient from COVID-19 infection, which in turn reduced distress related to COVID-19 infection. This is consistent with qualitative data that suggests young carers view social distancing restrictions as being protective [20]. The comparisons involving the OIFM group showed that higher levels of perceived insufficient home space during the second lockdown were correlated with greater fear of COVID-19 in the OIFM group, but the correlations between these two variables were not significant in the non-carer group. One explanation for this significant association is that greater fear of COVID-19 is likely to lead to young adult carers of ill non-parent family members spending more time at home to avoid infection, which may in turn leads to a sense of restlessness due to being restricted to the home and hence, perceptions of insufficient space. This proposal is supported by qualitative data obtained from young carers in the pandemic who report difficulties related to feeling cooped up with household members including the care-recipient [20].

Results showed that the COVID-19 Context Index was not significantly correlated with any outcome for the PI group. However, higher scores on the COVID-19 Context Index were related to poorer mental health across all but one outcome (increase in home violence) for the OIFM group and across five of the seven outcomes for the non-carer group. The absence of significant correlations between the COVID-19 Context Index and outcomes in the PI group may be due to the fact that we did not measure potentially important COVID-19 and lockdown risk factors that are more relevant to young adult carers in a parental illness context (e.g., access to external support for the ill parent, number of younger or older siblings, perceived seriousness of parental illness; [20]). The variation in relations between COVID-19 and lockdown risk factors and mental health outcomes between PI and OIFM young adult carers underscores the importance of distinguishing between these subgroups of young adult carers. Consistent with these findings, young carer studies that have included young adults have found that these two groups differ in relation to caregiving responsibilities and experiences [24] and adjustment outcomes [26].

As expected, young adult carers caring for an ill parent reported poorer mental health than non-carers across all seven mental health outcomes. Young adult carers of other ill family members also reported poorer mental health than non-carers, but across fewer outcomes (the general mental health outcomes of anxiety, depression, and wellbeing and one COVID-19 related outcome, increased family violence). These findings are consistent with prior young adult carer research that has shown that young adult carers report poorer mental health than non-carer peers [8,11]. The present study extends this body of research by showing that these differences are likely to be amplified in a pandemic because they extend to COVID-19 and lockdown related mental health outcomes. The nature of the COVID-19 mental health outcomes measured in this study and the fact that they are likely to be elevated in many young adult carers, raises grave concerns for the welfare of these vulnerable young people. For example, relative to non-carer peers, the marked increase in family violence reported by young adult carers in both parental illness and other ill family member contexts, suggests that during a pandemic, families with young adult carers should be prioritized with respect to assessment and intervention by healthcare professionals and domestic violence support services. In addition, as shown above and consistent with other pandemic studies, COVID-19 mental health risk factors add to the many challenges already inherent in informal caregiving [18].

Overall, the adverse mental health effects of caring for an ill parent were greater than the effects of caring for an ill non-parent family member. These findings are consistent with prior young carer research which shows that the presence of any family member with an illness is associated with greater risk of mental health difficulties for youths relative to peers from healthy families, and that this risk is elevated if the ill family member is a parent [2,26]. However, findings from the present study extend this body of evidence and show that this effect is also present for young adult carers and that it emerges across a range of mental health domains in the context of a pandemic.

Mirroring the above mentioned pattern of differences in mental health between young adult carers and non-carers, a greater proportion of young adult carers relative to non-carers reported clinically significant levels of anxiety and depressive symptoms. However, in the present study fewer young adult carers reported clinically significant levels of distress (19.00% anxiety symptoms, 28.63% depressive symptoms) compared to two prior young adult carer studies that assessed mental health problems (43.9% depressive symptoms, [11]; 39.23% anxiety and depressive symptoms, [8]). This divergence may be due to differences across studies with respect to methodologies (e.g., measurement and sampling), population (e.g., students vs. community), culture (e.g., North America and Norway vs. Italy), and healthcare systems. Interestingly, in the present study, relative to non-carers, higher proportions of young adult carers reported flourishing (29.25% vs. non-carers 18.28%) and fewer fell in the languishing band (21.88% vs. 25.04% non-carers). This pattern of findings regarding the categorizing of participants into the normative bands for the wellbeing measure should be viewed in the context of the results from analyses that examined the impact of PI, OIFM and non-carer status on wellbeing treated as a continuous variable. The latter showed that the two young adult carer groups had poorer wellbeing outcomes than non-carers. Considering these wellbeing findings together, although overall young adult carers report lower wellbeing than non-carers, it seems that a small but considerable proportion of young adult carers experience flourishing. This may occur in those who recognize the skills and other benefits gained from their caregiving role [9]. For example, young adult carers have reported benefits such as improved skills in organizing their educational and private lives and greater perceived maturity relative to non-carer peers [5,23]. Indeed, one study showed that 89.49% of young carers (including young adult carers) reported one or more benefits from their caregiving, and greater benefit finding was related to better mental health across multiple domains [54]. The only quantitative study of young adult carers that has assessed a positive indicator of mental health found that markedly fewer (30.28%) young adult carers reported high levels of life satisfaction compared to non-carers (41.40%) [8].

The elevated levels of mental health problems among many young adult carers indicate that they should be prioritized for mental health promotion interventions, particularly during a pandemic. A potentially important intervention target is psychological flexibility which has been found to buffer the adverse effects of caregiving and stress in young carers [55]. Enhancing psychological flexibility via acceptance and commitment therapy (ACT) has been shown to improve mental health in young adults [56]. In addition, a meta-analysis of ACT interventions for adult family carers found that ACT had small to moderate effects on anxiety, depression, stress and quality of life [57]. Findings from the present study delineate COVID-19 and lockdown mental health risk factors that can be used to identify at-risk young adult carers during the pandemic. Given the social restrictions used to manage the spread of COVID-19, flexible delivery of mental health interventions is critical for effective large-scale dissemination [58]. ACT has been flexibly delivered via group [59], online [56], and mobile app [60] modes. Given that caregiving is a profoundly interpersonal role, it is also important that young adult carers be resourced with the necessary practical and social supports via the provision of personal assistance to family carers and the ill family member [61,62].

The present study has several limitations. First, convenience sampling and the over-representation of female participants limit the generalization of findings. Second, the reliance on self-report measures increases the risk of common method variance. Third, level of caregiving was not assessed, and we were therefore unable to examine differences in caregiving across groups or the specific effects of COVID-19 and lockdown risk factors on the caregiving role of young adults. However, prior studies consistently show that young carers and young adult carers report higher levels of caregiving than non-carer peers [8,11,23,24], and preliminary data suggests the pandemic has exacerbated the caregiving burden of informal young and adult carers [18,20]. Fourth, as mentioned above, we examined a limited range of COVID-19 and lockdown risk factors, and therefore we may have omitted risk factors that are particularly potent for some young adult carers. Fifth, we did not assess all possible risky health behaviors and therefore some potentially important harmful health behaviors were not assessed (e.g., restrictive food intake). Sixth, we did not assess variations in family structure (e.g., single parent vs. dual parent families), which may affect the mental health of young adult carers. Finally, the cross-sectional study design precludes clarification of the causal directions among study variables. The strengths of this study include the assessment of a broad range of mental health outcomes, the inclusion of a positive mental health dimension (wellbeing), a sample of young adult carers drawn from the community, a relatively large sample size, the assessment of family health status (PI vs. OIFM), the contextualizing of young adult carer mental health in the COVID-19 pandemic and the use of a young adult age range that better reflects the duration of emerging adulthood in contemporary society within developed countries.

## 5. Conclusions

This is the first published quantitative study to investigate the mental health of young adult carers in the context of the COVID-19 pandemic. Findings from this study delineate key COVID-19 and lockdown mental health risk factors for young adult carers. A related study outcome is the development and preliminary testing of a COVID-19 Context Index, which is identical to a previously published measure of COVID-19 risk factors [13]. As predicted, results showed that, compared to non-carers, young adult carers reported poorer mental health across COVID-19-related and general mental health outcomes. Furthermore, results indicated that young adult carers caring for an ill parent reported poorer mental health than young adult carers of ill non-parent family members. Notably, the important, albeit minority of young adult carers who report clinically significant levels of distress in addition to the elevated levels of pandemic-related family violence, fear of COVID-19, risky health behaviors and loneliness among young adult carers in general, highlight this group as a priority for mental health support. Along with young adult carer focused mental health promotion interventions, a whole-of-family approach is needed to address family level issues, particularly domestic violence. Furthermore, young adult carers typically engage with a range of sectors (e.g., education, employment, vocational training, health, and welfare), which should be alerted to the needs of young adult carers and how these are exacerbated by the COVID-19 pandemic. Young adult and young carer targeted policies and supports across these sectors should be developed to promote mental health in this vulnerable and often hidden army of informal carers that make a critical unpaid caregiving contribution to the health care system.

## Figures and Tables

**Table 1:** Descriptive data on socio-demographics, caregiving context and COVID-19 context variables in the parental illness group (PI), other ill family member (OIFM) group, and non-carer group.

	PI (*n* = 268)		OIFM (*n* = 97)		Non-Carers (*n* = 1458)	
Variable	%	*M* (*SD*)	%	*M* (*SD*)	%	*M* (*SD*)
*Socio-demographics*						
Gender: female	74.25		72.16		71.26	
Age years		24.03 (2.72)		24.02 (2.80)		24.50 (2.86)
Education						
Primary school	2.24		3.09		4.05	
Secondary school	47.01		47.42		42.03	
Bachelor’s degree	46.64		44.33		49.93	
Postgraduate course	4.10		5.15		3.98	
Single	96.27		92.78		86.97	
Married or living with a partner	3.73		7.22		12.83	
Currently studying	65.67		62.89		56.24	
Currently working	25.00		28.87		37.72	
Not in education, employment, or training	10.82		11.34		9.19	
Socio-economic status						
<€15,000	19.78		15.46		16.60	
€15,001–€36,000	42.16		42.27		42.94	
€36,000–€70.000	29.85		32.99		29.29	
>€70.000	5.22		5.15		7.89	
Italian nationality	98.51		98.97		97.74	
Presence of a physical health condition	10.82		14.43		8.30	
*Family caregiving context variables*						
Ill mother	61.94					
Ill father	54.48					
Ill siblings			59.79			
Ill grandparents			43.30			
Physical illness	66.04		57.73			
Mental illness	46.64		52.58			
Insufficient home dimension ^a^		1.44 (1.02)		1.45 (1.12)		1.41 (1.02)
Working or studying from home ^a^		2.55 (1.84)		2.38 (1.84)		2.30 (1.86)
Currently in red zone	10.45		9.28		9.05	
COVID-19 infected	15.21		8.25		14.18	
COVID-19 hospitalized	0.00		0.00		0.00	
Family member infected	27.00		17.53		24.53	
Family member hospitalized	5.22		3.09		4.18	
Family member death	3.00		4.12		4.18	
Lost work	5.22		1.03		3.02	
Redundancy payment	1.49		3.09		1.99	
Substantial income reduction	4.85		3.09		4.12	

Note. ^a^ Variables coded on a 5-point Likert scale 0 = not at all or never to 4 = very much or always.

**Table 2:** Descriptive data on COVID-19-related mental health and general mental health outcomes in the parental illness group (PI), other ill family member (OIFM) group, and non-carer group.

	PI (*n* = 268)			OIFM (*n* = 97)			Non-Carers (*n* = 1458)		
	*M* (*SD*) or %	Range	α	*M* (*SD*) or %	Range	α	*M* (*SD*) or %	Range	α
*COVID-19-related mental health outcomes*									
Risky health behaviors ^a^	1.03 (0.53)	0–2.50	−	1.04 (0.61)	0–2.25	−	0.94 (0.50)	0–3	−
Loneliness	1.70 (0.86)	0–3	0.84	1.58 (0.89)	0–3	0.87	1.48 (0.90)	0–3	0.87
Increased home violence ^a^	0.64 (0.93)	0–4	−	0.53 (0.82)	0–4	−	0.31 (0.64)	0–4	−
Fear of COVID-19	12.30 (4.63)	7–35	0.85	12.23 (4.79)	7–27	0.85	11.41 (4.02)	7–32	0.82
*General mental health outcomes*									
Anxiety ^¥^	9.23 (4.95)	0–21	0.88	8.99 (5.34)	0–21	0.90	7.35 (4.90)	0–21	0.90
Normal	18.01%			22.11%			32.43%		
Mild	37.55%			36.84%			38.54%		
Moderate	27.20%			22.10%			17.89%		
Severe	17.24%			18.95%			11.14%		
Depression ^¥^	11.46 (5.93)	0–27	0.88	11.24 (6.35)	0–27	0.89	9.03 (5.65)	0–27	0.88
Normal	9.96%			15.79%			21.65%		
Mild	33.33%			30.53%			40.52%		
Moderate	26.82%			26.31%			20.51%		
Severe	29.89%			27.37%			17.32%		
Wellbeing ^¥^	31.15 (13.22)	2–61	0.91	31.59 (13.83)	0–60	0.91	35.12 (12.94)	0–70	0.90
Flourishing	27.64%			30.85%			18.28%		
Moderate	54.07%			50.00%			56.68%		
Languishing	18.29%			19.15%			25.04%		

Note. ^a^ Variables coded on a 5-point Likert scale 0 = not at all or never to 4 = very much or always. ^¥^ Mental health classifications are based on normative data described in the Measures subsection.

**Table 3:** Correlations between COVID-19 context variables and the mental health outcomes in the parental illness group (PI), other ill family member (OIFM) group, and non-carer group.

	***COVID-19-Related Mental Health Outcomes***											
	**Risky Health Behaviors**			**Loneliness**			**Increased Home** **Violence**			**Fear of COVID-19**		
	**PI**	**OIFM**	**Non-Carers**	**PI**	**OIFM**	**Non-** **Carers**	**PI**	**OIFM**	**Non-** **Carers**	**PI**	**OIFM**	**Non-Carers**
Insufficient home dimension	0.018	0.222 *	0.008	0.021	0.216 *	0.099 ***	0.113	0.204 *	0.102 ***	0.031	0.317 **^†^	−0.003 **^†^**
Working or studying from home	0.094	0.044	0.080 **	−0.045	0.123	0.104 ***	−0.108	0.170	0.028	0.003	−0.115	0.079 **
Currently in red zone ^b^	−0.117	0.033	0.006	−0.077	0.041	0.049	−0.105	0.103	0.002	−0.101	0.027	0.027
COVID-19 infected ^b^	0.045	−0.017	0.043	0.103	0.170 *	0.036	−0.041	0.015	0.027	−0.029	0.151	0.014
Family member infected ^b^	−0.051	0.027	0.027	0.171 **	0.088	0.015	−0.009	−0.035	0.03	−0.018	0.126	0.011
Family member hospitalized ^b^	−0.024	0.043	−0.007	−0.042	−0.017	0.033	−0.047	−0.026	0.000	0.009	0.037	0.014
Family member death ^b^	0.078	0.119	0.017	0.043	0.020	0.042	0.007	−0.152	−0.021	0.053	0.228 *	0.034
Reduced family income ^b^	0.035	0.099	0.027	0.130 *	0.190	0.036	0.033	−0.064	−0.001	0.057	0.114	0.041
COVID-19 Context Index	0.003	0.248 *	0.037	0.111	0.294 **	0.113 ***	0.069	0.174	0.095 ***	−0.010	0.417 ***^†^	0.018 **^†^**
	***General Mental Health Outcomes***											
	**Anxiety**				**Depression**				**Wellbeing**			
	**PI**	**OIFM**	**Non-** **carers**		**PI**	**OIFM**	**Non-** **carers**		**PI**	**OIFM**	**Non-** **carers**	
Insufficient home dimension	−0.033	0.278 **	0.079 **		−0.046	0.178	0.085 **		−0.119	−0.210 *	−0.122 ***	
Working or studying from home	0.121	0.062	0.174 ***		0.075	0.127	0.203 ***		−0.104	−0.139	−0.171 ***	
Currently in red zone ^b^	−0.163 **^†^	0.113	0.082 **^†^		−0.078 ^†^	0.028	0.103 ***^†^		0.120 ^†^	−0.063	−0.080 **^†^	
COVID-19 infected ^b^	−0.028	0.137	0.017		−0.008	0.178	0.004		0.006	−0.085	−0.002	
Family member infected ^b^	0.043	0.008	0.038		0.136 *	0.133	0.033		−0.030	−0.077	0.031	
Family member hospitalized ^b^	0.007	0.033	0.024		0.011	0.035	0.008		0.030	−0.042	−0.002	
Family member death ^b^	0.039	−0.034	0.056 *		0.056	0.025	0.041		0.078	0.045	−0.008	
Reduced family income ^b^	0.078	0.252 *	0.043		0.080	0.250 *	0.062 *		−0.072	−0.185	−0.066 *	
COVID-19 Context Index	−0.030	0.324 **	0.109 ***		0.012	0.281 **	0.117 ***		−0.080	−0.262 *	−0.118 ***	

Note. * *p* < 0.05, ** *p* < 0.01, *** *p* < 0.001. Significant correlations are displayed in bold. ^b^ Spearman’s correlations for categorical variables dummy coded as 1 = yes, 0 = no. ^†^ Corresponding pairs of correlation coefficients that significantly differ (*p* < 0.05 Bonferroni correction) between PI and non-carers or between OIFM and non-carers, based on Fisher’s Z transformations.

**Table 4:** Multivariate regressions predicting mental health outcomes.

	*COVID-19-Related Mental Health Outcomes*				*General* *Mental Health Outcomes*		
	Risky Health Behaviors	Loneliness	Increased Home Violence	Fear of COVID-19	Anxiety	Depression	Wellbeing
	*β* (ƒ^2^)	*β* (ƒ^2^)	*β* (ƒ^2^)	*β* (ƒ^2^)	*β* (ƒ^2^)	*β* (ƒ^2^)	*β* (ƒ^2^)
*Family caregiving status*							
**PI ^†^**	0.052 * (0.003)	0.073 ** (0.005)	0.161 *** (0.027)	0.067 ** (0.005)	0.114 *** (0.013)	0.125 *** (0.016)	−0.085 *** (0.007)
**OIFM ^†^**	0.042 (0.002)	0.019 (0.000)	0.070 ** (0.005)	0.042 (0.002)	0.068 ** (0.005)	0.078 ** (0.006)	−0.052 * (0.003)
*Confounders*							
COVID-19 Context Index	0.058 * (0.003)	0.136 *** (0.019)	0.096 *** (0.009)	0.043 (0.002)	0.118 *** (0.014)	0.130 *** (0.017)	−0.134 *** (0.018)
Age	−0.100 *** (0.010)	−0.087 ** (0.008)	−0.030 (0.001)	−0.024 (0.001)	−0.039 (0.002)	−0.078 ** (0.006)	0.041 (0.002)
Currently studying ^b^	0.064 (0.004)	−0.018 (0.000)	0.030 (0.001)	−0.026 (0.001)	0.062 (0.004)	0.020 (0.000)	0.034 (0.001)
Currently working ^b^	0.027 (0.001)	−0.113 ** (0.013)	0.021 (0.000)	−0.077 * (0.006)	−0.122 ** (0.015)	−0.158 *** (0.026)	0.204 *** (0.043)

Note. * *p* < 0.05, ** *p* < 0.01, *** *p* < 0.001. PI = parental illness, OIFM = other ill family member, *β* = standardized beta coefficient, ƒ^2^ = Cohen’s ƒ^2^. ^b^ Categorical variables dummy coded as 1 = yes, 0 = no. ^†^ The non-carer group was represented by a score of 0 on both PI and OIFM dummy variables, the standardized coefficient for PI indicates a test of the mean differences between PI and non-carers (controlling for the presence of OFMI), while the standardized coefficient for OIFM provided a test of the mean differences between OIFM and non-carers (controlling for the presence of PI).

## Data Availability

The dataset analysed during the current study are available from the corresponding author upon reasonable request.
